# Isolated angiitis of the central nervous system with tumor-like lesion, mimicking brain malignant glioma: a case report and review of the literature

**DOI:** 10.1186/1477-7819-9-97

**Published:** 2011-08-26

**Authors:** Gan You, Wei Yan, Wei Zhang, Shaowu Li, Guilin Li, Tao Jiang

**Affiliations:** 1 Department of Neurosurgery, Beijing Tiantan Hospital, Capital Medical University, Beijing 100050, China; 2 Department of Neuroradiology, Beijing Tiantan Hospital, Capital Medical University, Beijing 100050, China; 3 Department of Pathology, Beijing Neurosurgical Institute, Beijing 100050, China

**Keywords:** primary angiitis, vasculitis, tumor-like lesion, mimicking, glioma

## Abstract

**Background:**

Isolated angiitis of the central nervous system (IACNS) is a rare but severe vascular disease, which could present like an isolated inflammatory lesion on magnetic resonance imaging (MRI). To date, only a few such cases with tumor-like IACNS have been reported.

**Case Presentation:**

A 35-year-old woman presented with headache and left-sided weakness. MRI scans initially mislead us to a diagnosis of glioblastoma (GBM). Surgery was performed. The mass was sub-totally resected. Pathological examination confirmed a cerebral vasculitis. Radiological features, such as disproportionate mass effect, striped hemorrhage and abnormal enhancement of adjacent vessels, could be helpful to distinguish a tumor-like IACNS from a GBM. Single therapy with high doses of steroid did not improve the patient's condition. Combined therapy with prednisolone and cyclophosphamide showed great benefit to the patient. No relapse occurred during the period of 18 months follow-up.

**Conclusions:**

Although a tumor-like IACNS has no established imaging features, a diagnosis of tumor-like IACNS should be suspected when MRI shows inappropriate presentations of a tumor. Greater awareness of this potential manifestation of IACNS may facilitate more prompt diagnosis and treatment.

## Background

Isolated angiitis of the central nervous systmen (IACNS) represents a rare and poorly understood form of vascular inflammatory disease restricted to the brain and spinal cord. An average annual incidence rate of 2.4 cases per 1,000,000 person-years was found by a report from US [1]. Histopathology usually reveals granulomatous inflammation affecting arterioles and small arteries of the parenchyma and/or leptomeninges [2]. Non-specific clinical manifestations and various imaging findings often lead to an incorrect or delayed diagnosis and treatment [3], particularly for an extremely rare form of tumor-like lesion. In this report, we describe a woman with tumor-like IACNS that was initially mistaken for glioblastoma (GBM).

## Case Presentation

**Figure 1:**
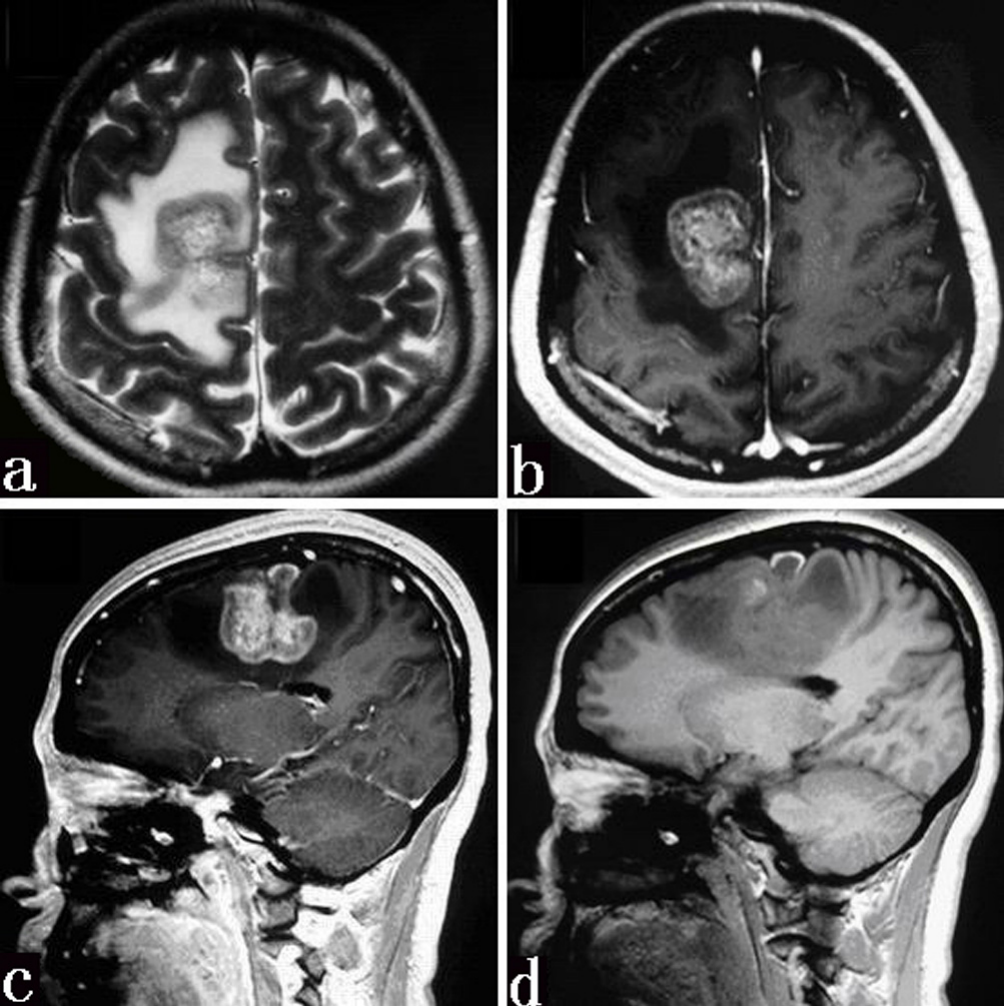
**Axial T2-weighted MR image (a) showing a mass with mixed signal intensity and a surrounding edema area**. On the T1-weighted image after the administration of contrast material (b and c), the mass has an inhomogeneous enhancement. Sagittal T1-weighted MR image without contrast (d) depicting a striped hemorrhage.

**Figure 2:**
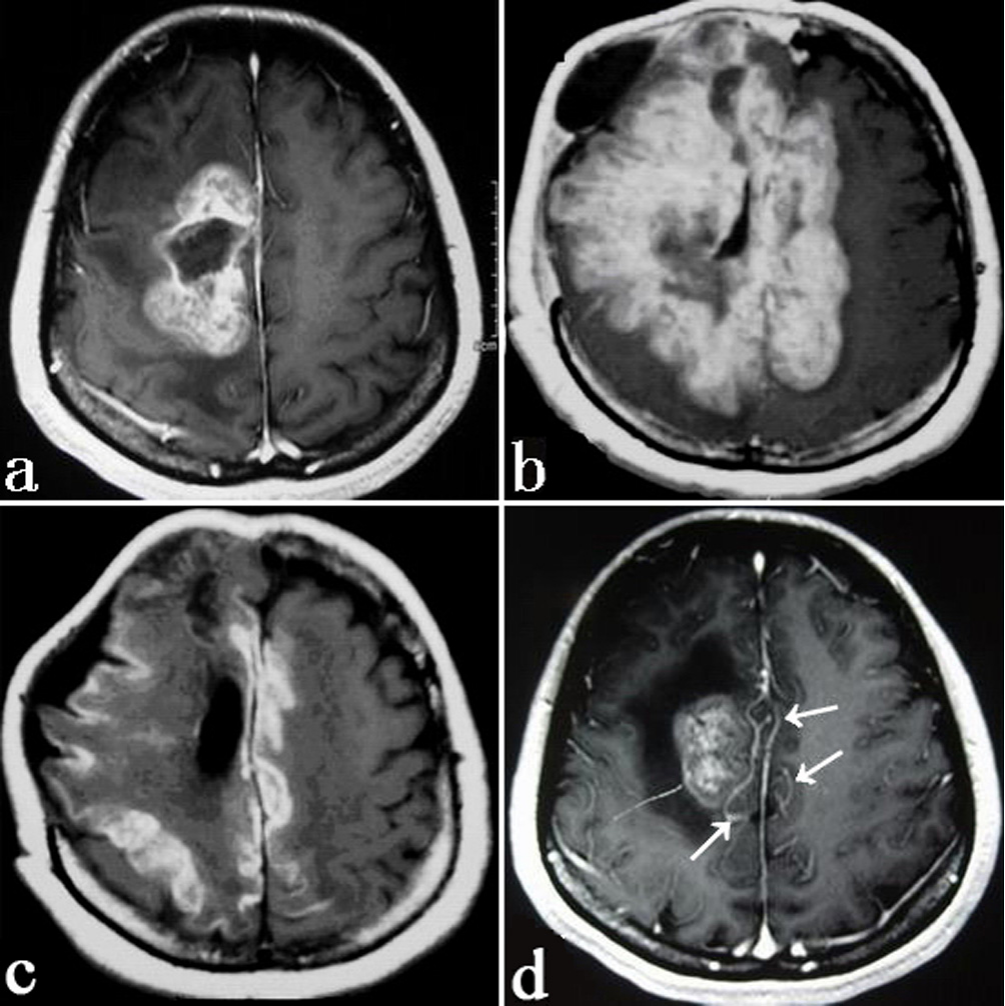
**Axial contrast-enhanced MR image showing a partial resection of the lesion**. (a) T1-weighted image showing the newly developed hyper-intensity diffusing to the opposite side (b). Great improvement after the treatment (c). T1-weighted MR images with contrast showing some abnormally enhanced vessels (arrows) (d).

**Figure 3:**
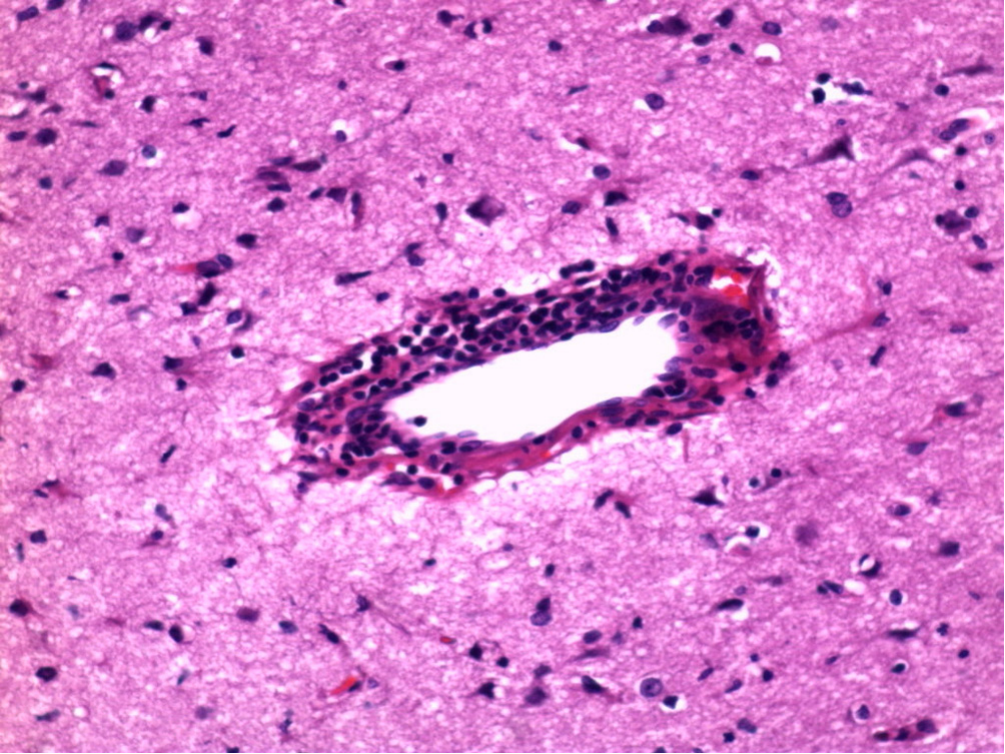
**Pathologic findings showing massive perivascular lymphocyte cuffing (HE stain, × 200)**.

In April 2008, a 35-year-old woman was admitted to our hospital due to headache and left-sided weakness over the preceding 1 month. The headache was diffuse and did not have a burning or stabbing sensation. The weakness of the left arm and jaw was mild. Her mental status was clear with normal orientation and alertness. It was negative in speech disorder and perceptual disturbance. A review of systems at the time of presentation revealed no additional symptoms, except for mild hypomnesis. There was no history of alcohol or illicit drug use. Toxic exposure history was negative. On neurologic examination, the patient presented moderate weakness on the left side. An equivocal Babinski sign was elicited in the left foot. Findings on physical examination were normal. Admission MRI study of the brain (Figure 1) revealed a tumor-like mass with edema and enhancement, which was initially suspected to be a malignant glioma. The patient subsequently underwent a craniotomy. Because a non-tumoral texture was palpated by the surgeon, the lesion was subtotally resectted (Figure 2a). Intraoperative pathologic examination showed no tumor cells but inflammatory cells and necrosis, which confirmed the surgeon's judgment. The patient's blood and cerebrospinal fluid (CFS) samples were collected at the end of operation. Concerned of a possible diagnosis of multiple sclerosis, a 3-day high-dose (1,000 mg daily) pulse therapy of methylprednisolone was initiated. Two weeks after surgery, the first postoperative MRI showed bilateral hyper-intensity in the frontal and parietal sub-cortex (Figure 2b). Results of the serum and CSF tests were as follows: Routine blood tests, as well as blood rheumatologic tests, blood immunoglobulins, blood antinuclear antibodies, antiphospholipid antibodies, and antineutrophil cytoplasmic antibody, were normal. Analysis of the CSF showed the increases of total protein (170 mg/dl; normal < 45 mg/dl) and myelin basic protein (MBP) (2.23 nmol/l; normal < 0.55 nmol/l). The immunoglobulin (Ig) G in CSF index was remarkably increased (20.5 mg/dl; normal < 6 mg/dl). Oligoclonal bands (OB) were negative. No marked increase in antiviral titers in the serum or CSF was observed. With great interests, we carefully rechecked all the paraffin sections of the mass, and a big surprise for us, we eventually found the evidence for cerebral vasculitis (Figure 3). Combined therapy with steroid and immunosuppressant was initiated immediately after the diagnosis as follows: intravenous prednisolone 1000 mg/d for 3 days, 500 mg/d for 3 days, 250 mg/d for 3 days, 125 mg/d for 3 days, followed by oral prednisolone 60 mg/d and cyclophosphamide 125 mg/d for 2 weeks. Six weeks after surgery, the patient's neurological symptoms gradually disappeared and repeated MRI confirmed remarkable improvement of affected brain (Figure 2c). She was discharged on a tapered dose of prednisolone and cyclophosphamide, and no relapse occurred during an 18-month follow-up.

## Discussion

IACNS, which was first reported as early as 1952 [4], can occur mostly when patients are 30 to 50 years old [3]. To date, a few such cases with tumor-like IACNS have been reported. Its etiology and pathogenesis are still unknown. It is very possible that viral infections initiate the inflammatory process that somehow becomes self-sustaining [5]. It is also speculated that there may be a genetic predisposition in certain individuals leading to an enhanced risk of a vasculitic process when there is an exposure to a particular antigen that "sets off" the immune system [6].

Clinical onset of IACNS is usually subacute, but it can have acute onset with rapid progress within a few days or weeks. The most frequent clinical manifestations at presentation are headache, altered cognition, hemiparesis or persistent neurological deficit or stroke [1]. Subarachnoid hemorrhage could be the first presentation of IACNS [7]. Less common complaints are aphasia, transient ischemic attack, ataxia, dysphasia, nausea or vomiting, loss of memory, seizure disorder, dyslalia, hypomnesia and paralysis. Interestingly, there have been so far two case studies reporting psychiatric symptoms in two patients with IACNS [8,9]. But all the symptoms above-mentioned are non-specific for diagnosis.

With regard to imaging characteristics, IACNS always presents a challenge in radiographic diagnosis. Its MRI findings are highly variable, ranging from multiple irregular white matter changes to intracerebral hemorrhages [7,10]. The present case demonstrated a large monofocal lesion with disproportionate mass effect, striped hemorrhage, and abnormal enhancement of adjacent vessels (Figure 2d) on MRI. Possibly some main artery and its branches were involved. So, given a tumor-like lesion with these characteristics on neuroimages, we suggest an IACNS should be considered in the differential diagnosis. This would be an important lesson learnt from this case. Several studies [3,11,12] to some extent emphasized the similar viewpoints. In addition, Campi et al. [13] indicated that VRPVS (dilatation of Virchow-Robin perivascular spaces), which signify severe but reversible perivascular inflammation causing blood-brain barrier disruption and injury of surrounding white matter, might likely be specific for vasculitis. Unfortunately, this did not show up in this case.

Moore [14] recommended a widely received standard for the diagnosis of IACNS in which he emphasized the importance of cerebral angiography and biopsy based on careful exclusion of other diseases. When cerebral angiography shows stenosis or occlusion of the cerebral vessels, brain biopsy should be performed [15]. However, the classic appearance of alternating narrowing and dilatation is not completely specific and has been observed in only 25% of patients with IACNS; the angiogram is normal in up to 40% of pathologically documented cases [16]. It should be individualized to make a differential diagnosis [3,14,17]. IACNS may clinically mimic encephalitis, multiple sclerosis, acute disseminated encephalomyelitis, myelinoclastic diffuse sclerosis, cerebral infarction, leukoencephalopathy or other brain tumors, when they present isolated lesions on MRI. It may be difficult to differentiate between IACNS and demyelinating or inflammatory diseases [18-20], because of similar symptoms, clinical exam and laboratory findings. Besides, it is also not easy to differentiate between IACNS and lymphoma when there are multiple-enhancing lesions with vasogenic edema on neuroimagines. Against the above, only biopsy allows a definite diagnosis. But sometimes, a single isolated negative biopsy does not necessarily exclude IACNS [21,22] or secondary CNS vasculitis. In that case, empirical treatment should be administrated.

No clinical trials have been performed in patients with IACNS, nor is it possible to draw firm conclusions from the current study because of the non-standardized nature of the treatment protocols used in these cases. Fountain et al. [23] reported a case with IACNS controlled by cyclophosphamide alone, while Carandang C and Grant A reported a female patient with IACNS responding to steroids alone [9]. But Barron et al. [24] indicated that steroid therapy alone failed to improve the condition. This finding is consistent with ours. Others recommended combination therapy consisting of prednisone and cyclophosphamide for at least 1 year [25,26]. In addition, Salvarani et al. [27] indicated that patients with IACNS which also have vascular deposits of β-peptide generally respond well to immunosuppressive treatment. However, the present case demonstrated good effects of immunosuppressant to patient without indication of amyloid protein deposition. Currently, most authors consent to combined and long-term therapy in treating IACNS. It is strongly suggested [1,28] that immunosuppressive therapy should be judiciously employed in patients with IACNS, based on the clinical features (any co-morbid conditions, the potential for neurological recovery, et al). In our case, the initial pulse therapy with steroid failed to prevent the rapid progress of vasculitis. Only combined therapy with cyclophosphamide improved her condition dramatically.

The prognosis of IACNS varies greatly and could be from months to years [1,11,17]. Untreated IACNS usually causes fatal outcome. Greater awareness of these potential manifestations of IACNS may facilitate more accurate diagnosis and prompt treatment.

## Conclusions

In conclusion, we suggest that correct diagnosis and appropriate treatment of tumor-like IACNS should be essential for prognosis. When the MRI shows a tumor-like subcortical lesion with disproportionate mass effect, and/or striped hemorrhage and/or abnormal enhancement of adjacent vessels, an IACNS should be include in the differential diagnosis. When high doses of steroids show no effect to the patient with tumor-like IACNS, combined treatment with cyclophosphamide followed by long term oral therapy is recommended.

## Consent

Written informed consent was obtained from the patient for publication of this Case report and any accompanying images. A copy of the written consent is available for review by the Editor-in-Chief of this journal.

## Competing interests

The authors declare that they have no competing interests.

## Authors' contributions

GY wrote the initial draft. WY drew the pictures. WZ revised the draft. SL described the MRI features. GL provided the pathological diagnosis. TJ was the surgeon and gave the final approval of the version to be published.
